# The mechanisms of tanshinone in the treatment of tumors

**DOI:** 10.3389/fphar.2023.1282203

**Published:** 2023-10-26

**Authors:** Pengyu Zhang, Wendi Liu, Yuan Wang

**Affiliations:** ^1^ The Medical College, Shandong University of Traditional Chinese Medicine, Jinan, China; ^2^ School of Chinese Medicine, Shandong University of Traditional Chinese Medicine, Jinan, China; ^3^ Department of Histology and Embryology, Shandong University of Traditional Chinese Medicine, Jinan, China

**Keywords:** tanshinone, cancer, molecular mechanism, traditional Chinese medicine, anti-tumor

## Abstract

Tanshinone is a lipophilic compound that is present in traditional Chinese medicine and is derived from the roots of *Salvia miltiorrhiza* (Danshen). It has been proven to be highly effective in combating tumors in various parts of the body, including liver carcinoma, gastric cancer, ovarian cancer, cervix carcinoma, breast cancer, colon cancer, and prostate cancer. Tanshinone can efficiently prevent the reproduction of cancerous cells, induce cell death, and inhibit the spread of cancerous cells, which are mainly involved in the PI3K/Akt signaling pathway, NF-κB pathway, Bcl-2 family, Caspase cascades, MicroRNA, MAPK signaling pathway, p21, STAT3 pathway, miR30b-P53-PTPN11/SHP2 axis, β-catenin, and Skp2. However, the properties and mechanisms of tanshinone’s anti-tumor effects remain unclear currently. Thus, this study aims to review the research progress on tumor prevention and mechanisms of tanshinone to gain new perspectives for further development and clinical application of tanshinone.

## 1 Introduction

Malignant tumors have had a crushing impact on human health and are identified as a main reason for death (Zuo et al., 2022). The global figures from 2020 revealed that there were around 19.30 million new cancer cases reported, resulting in approximately 10 million deaths. It is a matter of great concern that cancer incidence is increasing steadily, and by 2040, it is estimated to affect around 28.40 million people (Sung et al., 2021). Given this condition, significant focus has been placed on available treatments for malignant tumors. As a result, identifying the effective medical approach to treat malignant tumors has been of utmost importance to numerous researchers and clinical doctors.

**FIGURE 1:**
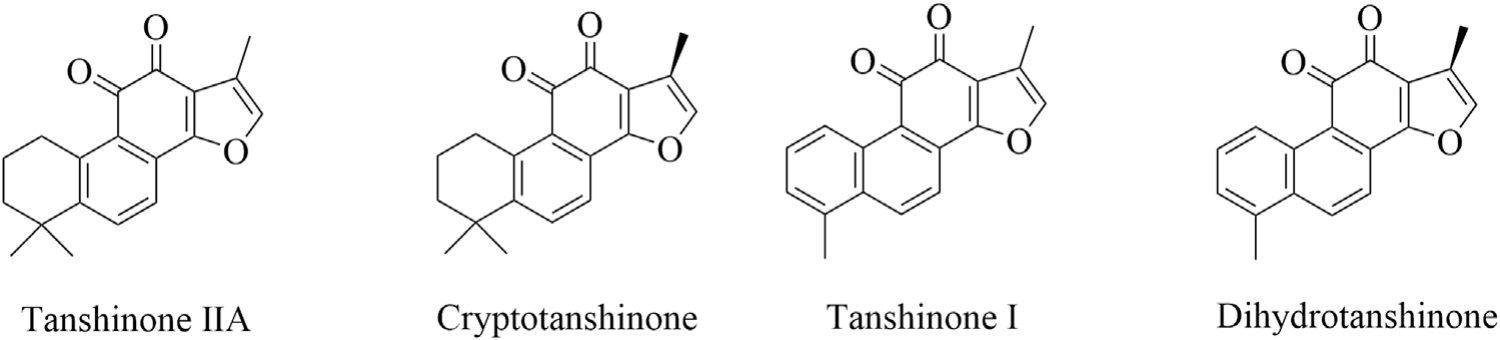
The chemical structural formula of tanshinone.

Danshen has been extensively researched as a natural active pharmaceutical ingredient since the 1930s (Li et al., 2009). According to the Chinese Pharmacopoeia’s 2020 Edition, *Salvia miltiorrhiza* is known for its ability to alleviate pain and eliminate blood stasis, as well as promote blood circulation, clear the heart, and reduce irritation. It contains both fat-soluble and water-soluble components that are effective. The fat-soluble components consist of tanshinone IIA, cryptotanshinone, tanshinone I, and dihydrotanshinone (Figure 1). While, the components that dissolve in water are salvianolic acid A, salvianolic acid B, purple oxalic acid, and rosmarinic acid. Among these two categories, the fat-soluble component, tanshinone, is highly valued due to its minimal toxicity, high effectiveness, safety, and ability to produce various pharmacological actions that are against tumors (Jin et al., 2021), inflammation (Ye et al., 2020), myocardial ischemia (Zhu et al., 2023), oxidative stress (Wu et al., 2021) and thrombosis (Feng et al., 2021), inhibit left ventricular hypertrophy (Pang et al., 2014), dilate blood vessels (Lu et al., 2021), resist atherosclerosis (Wen et al., 2020), protect brain tissue (Wang et al., 2022), improve microcirculation (Qin et al., 2023), significantly inhibit pulmonary fibrosis (Feng et al., 2020), and activate immunity (Chen et al., 2022).

Tanshinone has been shown to have anti-tumor properties among various common malignant tumors, including gastric (Ni et al., 2022), lung (Li et al., 2021), liver (Lee et al., 2010), breast (Wang et al., 2005) and colorectal (Jieensinue et al., 2018) cancers, which is achieved through various molecular mechanisms. Notably, the molecular mechanism behind the anti-tumor effect of continuous research has garnered significant attention, making it a research hotspot for numerous researchers. However, presently, there exists a requirement for a thorough review of the molecular mechanisms underlying tanshinone’s anti-tumor effects. Researchers have discovered that tanshinone primarily acts as an anti-tumor agent by promoting tumor cell death, inhibiting their proliferation, preventing migration and incursion, hindering immune evasion, and increasing the sensitivity of radiation and chemotherapy (Table 1). Thus, this article outlines the molecular mechanism of tanshinone in malignancies based on the five aforementioned aspects, in order to furnish scientific researchers and clinical workers with novel perspectives.

**TABLE 1:** Antitumor effect and mechanism of tanshinone.

Cancers	Effective concentrations	Real modules	Possible mechanism	Authors
Non-small-cell lung cancer	1.25, 2.5, 5, 10, 20, 40 μM	A549, PC9, H1299 and SPA-A1 cells	Downregulate the PI3K/Akt pathway, enhance Caspase 3 activity	Liao et al. (2019)
Glioblastomas	0.5, 1, 2, 5, 10, 25, 40 μM	GBM59, GL261 cells	Downregulate the PI3K/Akt/mTOR pathway	Schaf et al. (2023)
Ovarian cancer	1.2, 2.4, 4.8, 9.6 μg/mL	A2780, ID-8 cells	Inactivation of PI3K/AKT/mTOR pathway	Zhou et al. (2020a)
Prostate cancer	25, 50, 75, 100 μM	LNCaP, PC3 cells	Inactivation of PI3K/AKT/mTOR pathway, activate mitochondria intrinsic caspase cascade	Won et al. (2010)
Pancreatic cancer	1, 3, 9, 15, 30, 60 μg/mL	MiaPaCa-2 cells	Decrease the expression levels of EGFR, IGFR, and VEGFR, inhibit the PI3K/Akt/mTOR pathway	Su, (2018)
Gastric cancer	2.0, 3.7, 5.5 μg/mL	AGS cells	Decrease the expression levels of EGFR, IGFR, inhibit the PI3K/Akt/mTOR pathway	Su and Chiu, (2016)
Ovarian cancer	50, 100, 150, 200, 250 µM	A2780 cells	Attenuate PI3K/AKT/JNK pathway	Zhang et al. (2019b)
Breast cancer	20, 60 mg/kg	MDA-MB-231 cells	Decrease in NF-κBp65, increase in caspase-3 expression	Su et al. (2012)
Colon cancer	5, 20, 40 μM	HCT116, COLO205 cells	Decrease the NF-κB pathway and its downstream expression levels of COX-2, c-Myc, and Bcl-2	Bai et al. (2016)
Cholangiocarcinoma	10, 20, 40 μmol/L	HCCC-9810, RBE cells	Suppress the PI3K/Akt/NF-κB pathway, decrease the ratio of Bcl-2/Bax	Ke et al. (2017)
Breast cancer	1, 3, 10, 20, 30 μg/mL	MDA-MB-231 cell	Increase Bax to Bcl-xL ratios	Su and Lin, (2008)
Pancreatic cancer	4.2, 8.5 μM	BxPC-3 cells	Decrease protein expression of TCTP, MCL-1, and Bcl-xL	Huang et al. (2013)
Breast cancer	10, 20, 30, 40, 50, 60, 70, 80 μM	4T1 cells	Downregulate Bcl-2 and upregulate P53	Liu et al. (2023)
Colon cancer	6.25, 12, 20 μM	HCT116 cells	Release cytochrome c, stimulate caspase dependent pathways	Wang et al. (2015a)
Ovarian carcinoma	10, 20 μM	OVCAR3 and SKOV3 cells	Increase caspase-9, caspase-3, and PARP cleavage due to survivin suppression	Lin et al. (2015)
Myeloid leukemia cells	10, 20, 30, 40, 50 μM	K562 and HL 60 cells	Activate caspase-3 pathway linked to PI3K/Akt/survivin pathway	Liu et al. (2010)
chronic myelogenous leukemia	10, 20, 40, 80 μM	KBM-5 cells	Release cytochrome c, trigger caspase-3 and 9	Yun et al. (2013)
Non-small-cell lung cancer	5,10, 20, 40 μM	A549 and H292 cells	Target the circ_0020123/miR-1299/HMGB3 axis	Sun et al. (2023)
Osteosarcoma	N	U2OS and MG63 cells	Target the circ_0000376/miR-432-5p/Bcl-2 axis	Ye et al. (2022)
Acute leukemia cell	0.5, 1, 2 μM	HL-60 and THP-1 cells	Target the miR-497-5p/Akt3 axis	Nie et al. (2020)
Breast cancer	2.5, 5, 10, 20 μM	MCF7 cells	Target the miR-125b/STARD13 axis	Li et al. (2022a)
Colorectal cancer	N	SW480/R cells	Target the miRNA-30b-5p/AVEN axis	Ge and Zhang, (2022)
Melanoma	0.5, 1, 2, 4 μg/mL	A375 cells	Disrupt the PI3K-Akt-mTOR-p70S6K1 axis	Li et al. (2017)
Glioma	0.1, 10, 100, 1,000 ng/mL	U251 cells	Block the PI3K/Akt/mTOR signal pathway	Ding et al. (2017)
Acute promyelocytic leukemia	16, 32, 64 μmol/L	NB4 cells	Block the PI3K/Akt/mTOR axis and promote autophagy	Pan et al. (2021)
Acute myeloid leukemia	20, 40 μM	U937 cells	Block the PI3K/Akt/mTOR axis and promote autophagy	Zhang et al. (2019c)
Renal cell carcinoma	1, 5, 10, 30 μM	786-O and Caki-1 cells	Increase levels of LC3B, beclin-1, and Atg7	Kim et al. (2022)
Hepatocellular carcinoma	2, 4, 6 μM	HepG2, Huh7 cells	Inhibition of p53/DRAM-mediated autophagy	Liu and Liu. (2020)
Prostate cancer	3, 5, 10 μM	PC-3 cells	Increase beclin1 and LC3B expression and promote autophagy	Li et al. (2016a)
Glioblastoma	0.625, 1.25, 2.5, 5, 10 μM	U87 MG cells	Elevate LC3B and beclin-1 expression and promote autophagy	Jian et al. (2020)
Gastric cancer	2, 4 μM	BGC-823 and NCI-H87 cells	Cause ferroptosis via p53/SLC7A11 pathway	Guan et al. (2020)
Gastric cancer	25, 125, 250 μM	SGC-7901 and BGC-823 cells	Induce SLC7A11-mediated ferroptosis	Ni et al. (2022)
Head and neck squamous cell carcinoma	0.25, 0.5, 1 mg/L	FaDu cells	Decrease the ferroptosis gene FTH1	Mao et al. (2022)
Cervical cancer	2, 4, 8 μM	HeLa cells	Enhance pyroptosis by regulating the miR-145/GSDMD cascade	Tong et al. (2020)
Nasopharyngeal carcinoma	2, 4, 8 μM	HK1 cells	Enhance pyroptosis by modifying the miR 125b/foxp3/caspase-1 cascade	Wang et al. (2021)
Gastric cancer	1, 3, 9, 15, 15, 30, 60 μg/mL	AGS cells	Increase p-p38, p-JNK and p53, reduce p-ERK, CDC2 and Cyclin B1 expression	Su, (2014)
Breast cancer	1.5, 3, 4.5 μg/mL	BT-20 cells	Activate ER stress and MAPK pathway	Yan et al. (2012)
Prostate cancer	3, 6, 12 μM	PC3, LNCaP cells	Activate p38 MAPK pathway, inhibit GADD45A/PLK1 pathway	Wang et al. (2019a)
Prostate cancer	2.5, 5, 7.5, 10 μM	LNCaP cells	Activate p53/p21 signaling, inhibit AR	Won et al. (2012)
Melanoma	5, 10, 20 μM	A375 cells	Increase P21 and P53 expression, decrease Rb phosphorylation, Cdk2 and cyclin A2 expression	Zanre et al. (2022)
Gastric cancer	1, 5, 20 μg/L	SGC-7901 cells	Downregulate FOXM1, upregulate p21	Yu et al. (2017)
Osteosarcoma	0.08, 0.4, 2 μmol/L	U2OS, MOS-J cells	Suppress IL-6-induced JAK/STAT3 signalling pathway	Wang et al. (2019b)
Ovarian cancer	5, 10 μmol/L	Hey, A2780 cells	Suppress the STAT3/SIRT3 signaling pathway	Yang et al. (2018)
Gastric cancer	2.5, 5, 10 μg/mL	SNU-638, MKN1, AGS cells	Inhibit the phosphorylation of STAT3	Zhang et al. (2018)
Hepatocellular carcinoma	10, 20, 40, 80 μM	HepG2 cells	Regulate the miR30b-p53-PTPN11/SHP2 signaling pathway	Ren et al. (2017)
Liver cancer	0.5, 1, 2 µM	HepG2 cells	Reverse EGF and TGF-β1 mediated EMT via the PI3K/Akt/ERK pathway	Zhang et al. (2019a)
Colorectal cancer	0.5, 1, 2 µM	SW480 cells	Decrease MMP-9, Vimentin and increase E-cadherin levels	Zhang et al. (2016)
Prostate cancer	3, 6, 12 μM	PC3, LNCaP cells	Downregulate VEGF-1 and MMP-9 protein expression	Wang et al. (2019a)
Non-small-cell lung cancer	2.5, 5, 10 μg/mL	A549, NCI-H1299 cells	Inhibit the Cavin-1 mediated ERK/Smad2 pathway	Wang et al. (2023)
Colorectal cancer	2.5, 5, 10, 20 μM	HT-29 cells	Inhibit β-catenin/VEGF mediated angiogenesis	Sui et al. (2017)
Colon cancer	5, 10, 20, 50 μM	HC8693 cells	Suppress the COX-2-Wnt/β-catenin axis	Ma et al. (2018)
Gastric cancer	2.5, 5, 10 μg/mL	AGS, MGC-803 cells	Inhibit EMT by regulating miR874/HMGB2/β-catenin pathway	Yuan et al. (2020)
Colorectal cancer	1, 5, 10, 20, μM	SW480 cells	Promote the Mst1-Hippo pathway	Qian et al. (2018)
Liver cancer	5, 10, 20, 40, 80, 160 μM	Bel-7404, SMMC-7721 and Bel-7402 cells	Mediate SMAD7-YAP interaction to inactivate the TGF-β signaling pathway	Ma et al. (2019)
Cervix carcinoma	2, 4, 8 μM	Hela, C33A cells	Downregulate the expression of YAP	Qin et al. (2018)
Colon cancer	5, 10, 20, 30 μM	HCT 116 cells, HT29 cells	Downregulate the expression of Skp2, RhoA, and snail1	Lin et al. (2017)
Prostate cancer	5, 10 μM	DU145, 22Rv1, PC-3 cells	Downregulate the expression of Skp2, RhoA, and snail1	Wu et al. (2017)
Colorectal cancer	4, 8, 12, 16, 20 μM	HCT-116 cells	Reduce COX-2 and VEGF expression	Zhou et al. (2012)
Breast cancer	12.5, 25, 50 μM	MCF-7 cells	Attenuate HIF-1α accumulation and phosphorylation of Tyr705-STAT3	Wang et al. (2015a)
Colorectal cancer	2.5, 5, 10 μM	HCT-116 cells	Decrease HIF-1α, VEGF, and bFGF expression	Zhou et al. (2020b)
Colon carcinoma	0.5, 1, 2 mg/L	HT29 and SW480	Reduce uPA, MMP-2, and MMP-9 and increased TIMP-1, TIMP-2	Shan et al. (2009)
Colorectal cancer	6.25, 12.5, 25, 50, 100 μM	293T cells	Suppress IDO1 and TDO2, increase CD8^+^ levels	Zhang et al. (2022)
Non-small-cell lung cancer	N	H358-IR, H157-IR cells	Decrease expression of PPAT, elevate Caspase 3 and Caspase 8 levels	Yan et al. (2018)
Gastric cancer	2, 5, 10 μM	SNU-719R, SNU-620 cells	Suppress the expression of MRP1	Xu et al. (2018)
Colon cancer	6.25, 12.5, 25 μM	SW620 cells	Downregulate P-gp mRNA and protein levels	Hu et al. (2014)
Breast cancer	0.1, 0.5, 1, 5 mg/L	MCF-7 cells	Downregulate ABC transporters (P-gp, BCRP, and MRP1)	Li et al. (2019)

## 2 Tanshinone induces tumor cell death

### 2.1 Tanshinone induces tumor cell apoptosis

#### 2.1.1 PI3K/Akt signaling pathway

PI3K is imperative in activating Akt, which is essential for enhancing cellular survival and proliferation (Liu et al., 2009; Santarpia et al., 2012). The PI3K/Akt axis is a commonly dysregulated kinase cascade in human cancer and is activated by growth factor receptors (McCubrey et al., 2011). Research on non-small-cell lung cancer revealed that when the quantity of tanshinone IIA increased, the viability of A549 cells was reduced (Liao et al., 2019). Meanwhile, tanshinone IIA intervention had a concentration-dependent impact on the suppression of PI3K and downstream Akt protein phosphorylation. Nevertheless, the expression of Caspase 3, a protein that aids in cell apoptosis, significantly increased. Combining these findings, tanshinone IIA stimulated cell apoptosis by modulating the PI3K/Akt pathway and influencing Caspase 3 activity.

Schaf et al. (2023) reported the tanshinone IIA’s role in glioblastomas and the induction of apoptosis on GBM59 cells in a manner dependent on concentration. During that study, TSA treatment resulted in a reduction in the phosphorylated PI3K levels within the nucleus of glioblastoma cells, as well as a decrease in levels of AKT and mTOR regulated by PI3K. This suggested that tanshinone IIA effectively inhibited the multiplication of GBM59 cells and triggered their cell death, possibly due to the reduction of the PI3K/AKT/mTOR pathway. Another bioactive component tanshinone IA significantly induced apoptosis and promoted autophagy *in vitro and in vivo* by inactivating the PI3K/AKT/mTOR pathway in ovarian cancer (Zhou et al., 2020a). Consistent with the study above, Tan IIA appears to suppress the PI3K/AKT survival pathway, which aids in apoptosis induction signaling. According to a study conducted by Won et al. (2010), the levels of PI3K p85 subunit expression, AKT phosphorylation, and mTOR in LNCaP cells were decreased following Tan IIA treatment. Additionally, Tan IIA treatment triggered the mitochondria to release cytochrome c into the cytoplasm, while suppressing the expression of Mcl-1L and increasing levels of caspases-9 and 3, as well as PARP. Taken together, this study indicated that Tan IIA’s stimulation of apoptosis involved the activation of caspase cascades within the mitochondria and the suppression of the PI3K/AKT/mTOR axis.

Additional research into the mechanism revealed that tanshinone IIA hindered the PI3K/AKT/mTOR signaling pathway by diminishing levels of EGFR, IGFR, and VEGFR (Su, 2018). It was the initial study to show that Tan IIA hindered the growth of MiaPaCa-2 pancreatic cancer cells by reducing levels of EGFR, IGFR, and VEGFR, while also suppressing the PI3K/Akt/mTOR axis. Consistently, tan IIA in gastric carcinoma could substantially and concentration-dependently reduce EGFR, IGFR, PI3K, AKT, and mTOR protein expression both *in vitro and in vivo* (Su and Chiu, 2016). This report first unveiled tan IIA’s potential to inhibit gastric cancer via blocking the PI3K/Akt/mTOR pathway and lowering the EGFR and IGFR protein levels in AGS cell xenograft tumors.

JNK is activated by phosphorylation and triggered by cytokines, growth factors, or stress (Wagner and Nebreda, 2009). In a study on ovarian cancer, it was discovered that Tan-IIA deactivated the PI3K/AKT/JNK pathway to activate caspase-3, caspase-8, caspases-9 and downregulate Bcl-w, Mcl-1L, which led to cell apoptosis (Zhang et al., 2019b). It was also revealed that PI3K overexpression canceled out the influence of Tan IIA on AKT and JNK expression, and prevented ovarian cancer cells from apoptosis that Tan IIA induced. This suggested that Tan IIA’s anti-tumor activity might be linked to its suppression of the PI3K/AKT/JNK axis.

#### 2.1.2 NF-κB signaling pathway

The NF-κB transcription factor is a well-established regulatory protein that serves a crucial role in preventing cell apoptosis (Verzella et al., 2020). Research on breast cancer has revealed the viability of MDA-MB-231 was reduced with an increased concentration of tanshinone IIA (Su et al., 2012). Meanwhile, intervention with tanshinone IIA suppressed NF-κB p65 and increased Caspase 3 level concentration-dependently. After analyzing this study, tanshinone IIA possessed the capacity to trigger cellular apoptosis through the modulation of the NF-κB pathway and the activation of Caspase cascades.

Another study reported tanshinone IIA’s function in colon cancer and found it suppressed the growth of HCT116/COLO205 cells in a manner dependent on dose (Bai et al., 2016). In that study, the use of TSA reduced the NF-κB regulated gene expression levels, including COX-2, c-Myc, and Bcl-2, as well as the amount of phosphorylated NF-κB p65 in the nucleus of colon cancer cells. Additionally, another bioactive component cryptotanshinone was found to significantly induce apoptosis of HCCC-9810 and RBE cells by inactivation of the NF-κB pathway in cholangiocarcinoma (Ke et al., 2017). During this study, it was discovered that decreasing PI3K and Akt activity resulted in the deactivation of the NF-κB pathway and the alteration of the Bcl-2/Bax ratio. These indicated that tanshinone can significantly trigger the apoptosis of cancerous cells, which might be related to the decrease of anti-apoptotic proteins mediated by the NF-κB signaling pathway.

#### 2.1.3 Bcl-2 family

Bcl-2 is a molecule that can lessen caspase cascade reactions triggered by cytochrome c and prevent mitochondria from releasing pro-apoptotic proteins, ultimately serving as an anti-apoptotic agent (Straten and Andersen, 2010). Bcl-2, therefore, is crucial for the treatment of tumors. The research found that tanshinone IIA could promote MDA-MB-231 cell death in breast cancer via elevating the pro-apoptotic protein Bax and lowering the anti-apoptotic protein Bcl-2 levels (Su and Lin, 2008).

TCTP possesses an anti-apoptotic property that can be associated with the binding of MCL-1 and Bcl-xL (Zhang et al., 2002; Graidist et al., 2004; Liu et al., 2005), and the antagonization of Bax (Yang et al., 2005). In a pancreatic carcinoma study, it was found that the increased concentration of Tan IIA led to an increase in levels of Bax and Caspase-3, while the decrease of TCTP, Mcl-1, and Bcl-xL, thus inducing BxPC-3 cells cell apoptosis (Huang et al., 2013). During this process, the decrement of BXPC-3 cell activity and the increase of toxicity exhibited a direct correlation with time and dosage. Overall, Tan IIA has the potential to trigger apoptosis in pancreatic carcinoma by inhibiting the anti-apoptotic proteins of TCTP, Mcl-1, Bcl-xL and initiating the Caspase pathway’s activation.

The mutation or deactivation of p53 and the bcl-2 overexpression are frequently present in malignant tumors (Vousden and Lane, 2007). A study has reported Tan IIA promoted apoptosis in breast cancer when 4T1 cells were exposed to it at varying concentrations for 24, 48, and 72 h (Liu et al., 2023). It was found that Tan IIA could significantly suppress proliferation and facilitate apoptosis, concurrently resulting in the reduction of Bcl-2 expression and upregulation of phosphorylated p53 and Bax. To conclude, this shows that Tan IIA serves as an anti-neoplastic agent in breast cancer by controlling the levels of suppressor gene p53 and apoptotic factor Bcl-2.

#### 2.1.4 Caspase pathway

Caspases, endoprotease family, control cell death and inflammation. After signaling events, enzymes activate substrates and create a signaling cascade to induce apoptosis. A study in colon cancer discovered that dihydrotanshinone I-induced AIF increase significantly upregulated mitochondrial cytochrome c release in HCT116 cells (Wang et al., 2015a). At the same time, cytochrome c release stimulated caspase cascades that contributed to dihydrotanshinone I’s anti-colon cancer efficacy *in vitro and in vivo*. Additionally, Chiu et al. found that tan IIA increased ER stress by raising PERK, IRE1α, caspase-12, and ATF6 expression in BxPC3-derived xenograft tumors. These proteins caused eIF2α, and CHOP overexpression, leading to lowered Bcl-2 expression and enhanced caspase-3-mediated apoptosis *in vivo* (Chiu and Su, 2017). This finding indicated that tan IIA stimulated caspase cascades via ER stress, becoming a promising candidate for pancreatic cancer treatment.

A study in ovarian carcinoma revealed that tan IIA concentration-dependently increased caspase-9, caspase-3, and PARP cleavage due to survivin suppression (Lin et al., 2015). In this process, the effect of TRAIL was enhanced by tanshinone IIA through the downregulation of survivin in OVCAR3 and SKOV3 cells. Furthermore, Tan I, according to Liu et al. (2010), promoted apoptosis of myeloid leukemia cells via activating the caspase-3 cascade which might be associated with inactivation of the PI3K/Akt/survivin pathway. Similarly, during chronic myelogenous leukemia, tanshinone IIA decreased mitochondrial membrane potential (MMP), released cytochrome c, and triggered caspase-3 and 9, confirming mitochondria-dependent apoptosis (Yun et al., 2013). The Caspase cascade involved the increase of JNK and p38 phosphorylation expression in KBM 5 cells. In addition, tan IIA decreased hepatocellular carcinoma growth in a J5 xenograft animal model by boosting Bax and caspase 3 and lowering CD31 expression *in vivo* (Chien et al., 2012). Overall, tanshinone, according to different signaling events, modulates caspase expression to enhance the therapy of cancerous diseases.

#### 2.1.5 miRNA

MicroRNA (miRNA) encompasses a group of non-coding, single-stranded RNA molecules that exert a pivotal part in the initiation and advancement of tumors (Zhang et al., 2017; Tang et al., 2018). According to research, the upregulation of miR-125 expression may potentially augment the stemness of breast cancer cells. Nevertheless, tanshinone IIA therapy reduced cell viability and raised the propensity for apoptosis in MCF-7 cells (Li et al., 2022a). Meanwhile, miR-125b expression was downregulated whereas STARD13 expression was upregulated. Additionally, study found that tanshinone IIA effectively impeded the growth of breast cells by modulating STARD13 expression, which was achieved through the decrease of miR-125b. This study is the first to propose that STARD13 contributes to the development of breast cells, in conjunction with the miR-125b/STARD13 axis.

Similarly, Ge et al. conducted research to determine if tanshinone IIA’s effects on colorectal cancer were due to the miRNA/AVEN axis (Ge and Zhang, 2022). Apoptosis and caspase activation inhibitor (AVEN) exerts a cancer-facilitating part in tumors (Choi et al., 2006; Eissmann et al., 2013; Han et al., 2015a; Baranski et al., 2015). An *in vitro* study showed that tanshinone IIA could concurrently suppress SW480/R cell proliferation by targeting miR-30b-5p and enhance apoptosis by targeting AVEN in colorectal cancer. The study showed that tanshinone IIA treatment significantly increased miR-30b-5p expression in SW480/R cells, and miR-30b-5p suppression fully negated the inhibition of SW480/R cells’ malignant behaviors using tan IIA. While this was happening, AVEN expression decreased whereas activities of cleaved-caspase 3, Bax enhanced concurrently. These results indicated that tanshinone IIA induced apoptosis in colorectal cancer by increasing miR-30b-5p and decreasing AVEN.

**FIGURE 2:**
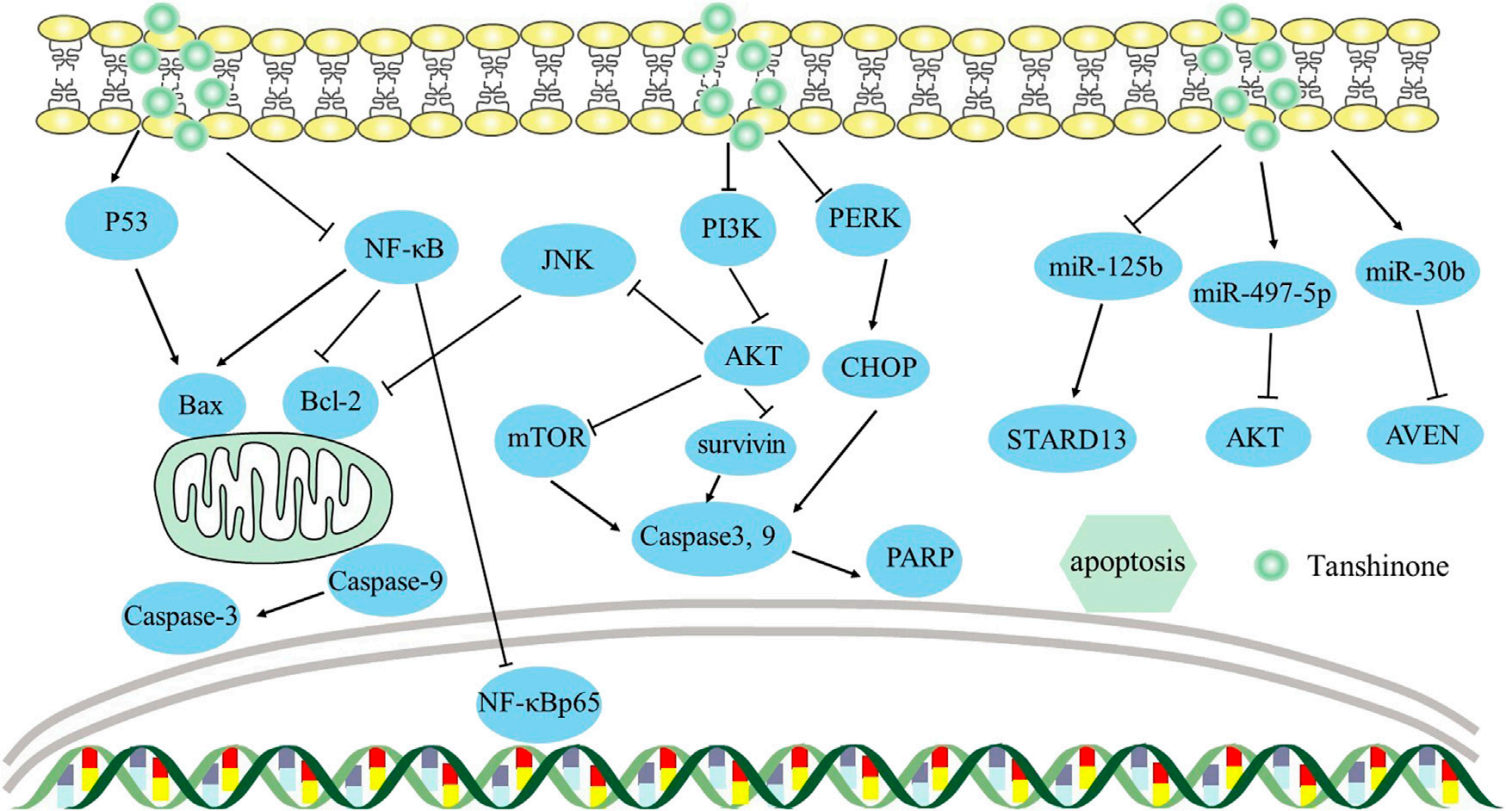
Mechanism of Tanshinone inducing apoptosis in tumor cells. Contents are as follows. Bcl-2 family: PI3K/AKT/JNK axis decreases Bcl-2 levels; NF-κB elevates Bax and decreases Bcl-2 levels; P53 elevates Bax. Caspase cascades: PI3K/Akt/survivin elevates Caspase-3 and Caspase-9 levels; PI3K/AKT/mTOR elevates Caspase-3 and Caspase-9 levels; PERK causes CHOP overexpression, leading to caspase-3-mediated apoptosis by ER stress. miRNA: miR-125b/STARD13 axis; miR-30b/AVEN; miR-497-5p/AKT.

In non-small-cell lung cancer, apoptosis of A549 and H292 cells was facilitated by tanshinone IIA through the circ_0020123/miR-1299/HMGB3 axis, which raised miR-1299 expression while decreased circ_0020123 and HMGB3 expression (Sun et al., 2023). Similarly, in osteosarcoma, tanshinone I induced apoptosis of U2OS and MG63 cells targeting the circ_0000376/miR-432-5p/Bcl-2 axis, lowering circ_0000376 and Bcl-2 and elevating miR-432-5p (Ye et al., 2022). This was the first study to reveal the connection between miR-432-5p and circ_0000376 or Bcl-2. Additionally, Nie et al. (2020) found that tanshinone IIA affected acute leukemia cell viability via the miR-497-5p/Akt3 axis, elevating miR-497-5p and lowering Akt3. This study was the first to reveal that tan IIA controlled AML growth by upregulating miR-497-5p. Overall, circRNAs and miRNAs function as mediators in the progression of malignancies, providing novel avenues for cancer therapy. The mechanism by which it induces apoptosis can be viewed in Figure 2.

### 2.2 Tanshinone induces tumor cell autophagy

In various cancer models, autophagy has two opposing effects. Autophagy has a cytoprotective impact in several cancer models but it could also convert from cytoprotective to cytotoxic autophagy in specific circumstances (Levy et al., 2017). Recently, Li et al. (2017) discovered that Tanshinone IIA reduced melanoma growth by disrupting the PI3K-Akt-mTOR-p70S6K1 axis and activating autophagocytosis in A375 cells. Similarly, Ding et al. (2017) found that tan IIA blocked the PI3K/Akt/mTOR pathway and lowered p-PI3K and p-Akt levels in U251 glioma cells, inducing autophagy and inhibiting cell viability. In acute promyelocytic leukemia, tan IIA blocked the PI3K/Akt/mTOR axis and promoted autophagy of NB4 cells, decreasing PI3K, Akt, and mTOR protein levels while raising p-ULK-1 and LC3B levels (Pan et al., 2021). Similar to these results, tan IIA inhibited PI3K/Akt/mTOR to trigger acute myeloid leukemia U937 cell death and autophagy *in vitro and in vivo* (Zhang et al., 2019c). In conclusion, these findings suggested that tan IIA could block the PI3K/Akt/mTOR axis to induce autophagy in cancerous cells. Additionally, Kim et al. (2022) found that tanshinone IIA blocked β-catenin translocation into the nucleus, leading to increased levels of autophagy-related genes (LC3B, beclin-1, Atg7) and the formation of autophagosomes in 786-O and Caki-1 cells, further inducing renal cell carcinoma cells autophagic death. Conversely, a study in hepatocellular carcinoma found that inhibition of p53/DRAM-mediated autophagy resulted in lower expression of LC3B and beclin-1, ultimately inducing cell death (Liu and Liu, 2020). Furthermore, in comparison to Tanshinone I alone, the autophagy inhibitor 3-MA dramatically reduced cell proliferation and viability in HepG2 and Huh7 cells. Taken together, autophagy has a contradictory effect on distinct cancer cell types, suggesting it might be a cancer therapy target.

Furthermore, according to many studies, tanshinone-induced cell autophagy involves ROS accumulation in many malignancies. Li et al. (2016a) observed that tanshinone IIA induced PC-3 cell autophagy by increasing beclin1 and LC3B expression in prostate cancer. In this process, tanshinone IIA-induced ROS was vital for autophagy start, while ROS scavenger NAC decreased beclin1, LC3B, and cleaved caspase-3 expression. Similarly, tanshinone I triggered ROS-dependent autophagy in glioblastoma, elevating LC3B and beclin-1 expression significantly in U87 MG cells (Jian et al., 2020). Taken together, these results showed that tanshinone-induced ROS was essential for autophagy initiation.

### 2.3 Tanshinone induces tumor cell ferroptosis

Ferroptosis is a non-apoptotic cell death that involves iron-dependent lipid peroxidation (Mou et al., 2019). At present, tan IIA’s ferroptosis mechanism against cancer has been identified, providing significant insight into its application in cancer intervention. In gastric cancer, tanshinone IIA caused BGC-823 and NCI-H87 cells ferroptosis via the p53/SLC7A11 pathway (Guan et al., 2020). It concentration-dependently upregulated p53 and inhibited xCT, lowering intracellular GSH and cysteine and increasing ROS levels to inhibit the growth of gastric cancer. Similarly, tanshinone IIA suppressed gastric cancer cell stemness via SLC7A11-mediated ferroptosis (Ni et al., 2022). The proportion of subpopulation CD44^+^ cells, a tumor stemness marker, was inhibited during this process. As a consequence, ferroptosis has been regarded as a prospective candidate for therapeutic intervention in cancer stem cells.

Additionally, tan IIA treatment significantly lowered the survival and invasiveness of FaDu cells by decreasing the ferroptosis gene FTH1, which is widely expressed in head and neck squamous cell carcinoma (Mao et al., 2022). This study also revealed that due to cancer cells’ high iron levels and higher sensitivity to ferroptosis, ferroptosis may be a potential cancer therapy.

### 2.4 Tanshinone induces tumor cell pyroptosis

**FIGURE 3:**
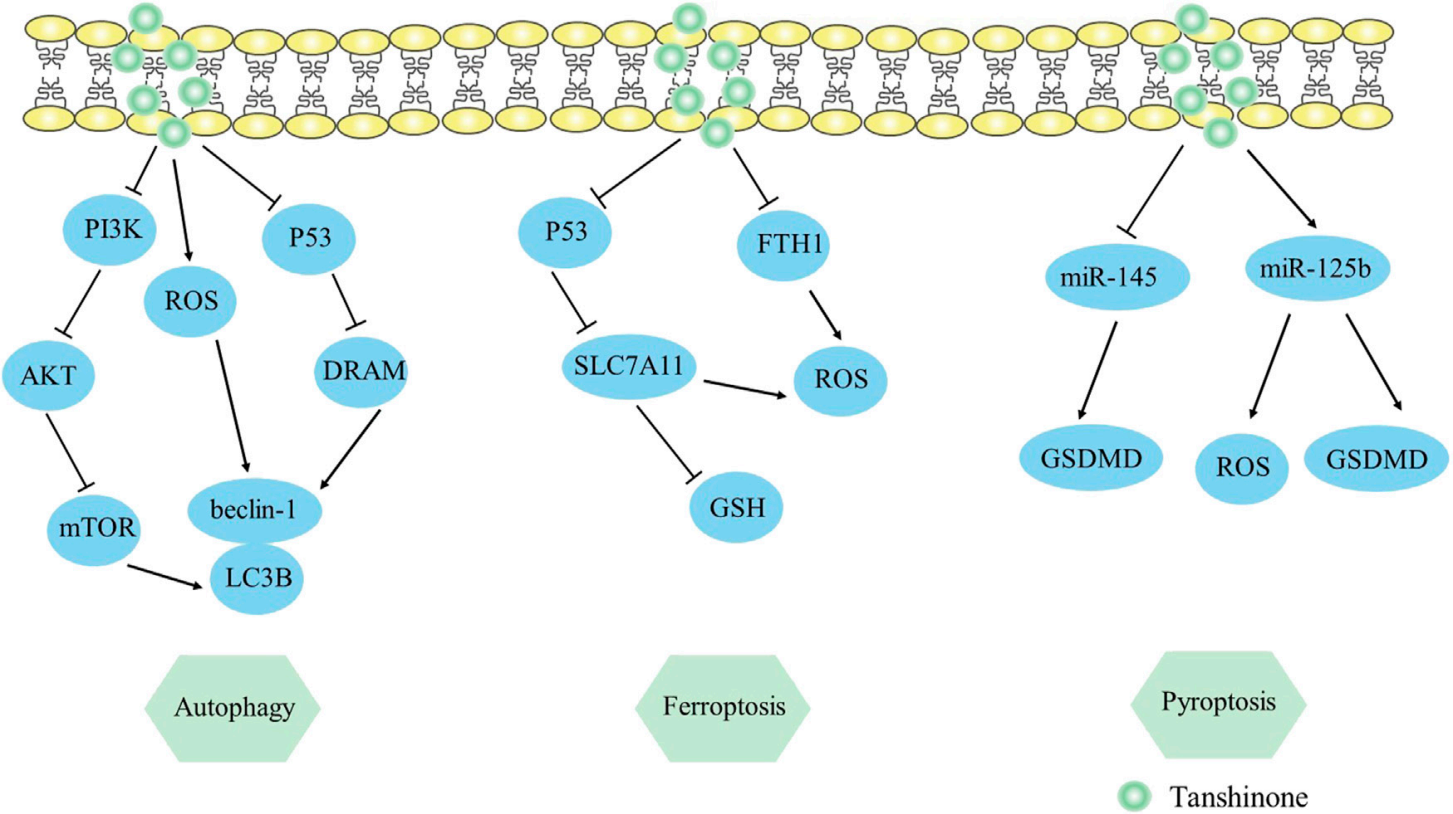
Mechanism of Tanshinone inducing autophagy, ferroptosis, pyroptosis in tumor cells. Contents are as follows. Autophagy: PI3K/AKT/mTOR elevates LC3B, beclin-1 levels; ROS elevates LC3B, beclin-1 levels; P53/DRAM mediates autophagy. Ferroptosis: P53/SLC7A11 pathway elevates ROS and decreases GSH expression; Decrease the ferroptosis gene FTH1. Pyroptosis: Regulate the miR-145/GSDMD cascade; miR-125b elevates GSDMD and ROS levels.

Pyroptosis is a type of programmed cell death characterized by inflammation, which is correlated with the NLRP3 inflammasome (NLRP3, ASC, and caspase-1) and the release of pro-inflammatory proteins (IL-18, IL-1β) (Li et al., 2022b). Notably, gasdermin D (GSDMD) serves as the principal mediator of pyroptotic cell death. Tong et al. (2020) observed that tanshinone IIA exhibited the capacity to enhance pyroptosis in HeLa cells by regulating the miR-145/GSDMD cascade, causing a decrease in cell proliferation. This study was the first to link miR-145 to pyroptosis and consider miR-145 as a therapy target for cervical cancer. In addition, Wang et al. (2021) discovered that pyroptotic levels were raised in nasopharyngeal carcinoma by modifying the miR- 125b/foxp3/caspase-1 cascade. Tanshinone IIA increased GSDMD and caspase-1 expression, raised ROS and LDH levels, and upregulated IL-18 and IL-1β, thus triggering the pyroptosis of HK1 cells. Collectively, studies of pyroptosis might provide vital insights on the utilization of tan IIA in cancer therapy. The mechanism by which it induces pyroptosis can be viewed in Figure 3.

## 3 Tanshinone prevents tumor cells from proliferating

Malignant tumors often exhibit aberrant cell proliferation as one of their defining characteristics (Hanahan and Weinberg, 2011). In clinical practice, preventing the growth of tumor cells has proven to be an effective strategy in treating tumors.

### 3.1 MAPK signaling pathway

MAPK is classified as a serine/threonine kinase and is activated by various signals to enhance cell survival, proliferation, and apoptosis (Molina and Adjei, 2006). The MAPK pathway contains ERK, p38, and JNK. The p38 MAPK and the JNK MAPK pathways have been associated with anti-proliferative functions, but the ERK MAPK pathway appears to exert opposing effects (Nordstrom et al., 2009). According to the report, tan IIA boosted the production of p-p38 and p-JNK, while p-ERK expression was decreased. Meanwhile, the activation of p53 led to a rise in p21 level, which in turn decreased CDC2 and cyclin B1 expression, and triggered AGS cells to be arrested in the G2/M phase (Su, 2014). This indicated that Tan IIA likely caused the cell cycle to stall in gastric cancer by upregulating p-p38, p-JNK, p53, and p21, downregulating CDC2 and cyclin B1 levels. Consistently, Yan et al. (2012) observed that tanshinone IIA inhibited BT-20 human breast cancer cells’ ability to proliferate by activating ER stress (upregulating caspase-12, CHOP levels and downregulating Bcl-2 expression) and MAPK pathway (upregulating p-p38, p-JNK protein expression and downregulating p-ERK expression).

Another study on prostate cancer revealed that tanshinone analog treatment led to a reduction in survival and proliferation, possibly due to the increase in p38 and p53 proteins (Wang et al., 2019a). Subsequently, p38 and p53 respective downstream pathways such as cyclin b1/CDC2 and GADD45A/PLK1 were inactivated, inducing G2/M arrest in the two PCa cells. This research indicates that p38/cyclin b1/CDC2 and p53-dependent GADD45A/PLK1 pathways are two promising therapeutic strategies for inhibiting proliferation.

### 3.2 p21 protein

P21, a CDK inhibitor, modifies the G1 or S phases of the cell cycle (Roskoski, 2019). It plays a crucial role as a downstream target of p53, facilitating the growth-inhibitory consequences of p53 in neoplastic formations (el-Deiry et al., 1993). Given the important function of p53 in regulating cell cycle progression and the impact of p21/WAF1 proteins as CDK inhibitors, research regarding pertinent mechanisms has been conducted. Won et al. (2012) discovered that tanshinone IIA induced G1 stagnation by activating p53/p21 signaling and inhibiting androgen receptors in LNCaP cells. Furthermore, tanshinone IIA induced the cell cycle in prostate cancer to stall at the G1 phase by lowering cyclin D1, CDK2, and CDK4. Another research found that treatment with cryptotanshinone could potentially enhance P21 and P53 expression in melanoma (A375 cells), and reduce Rb phosphorylation, CDK2 activation, and cyclin A2 expression (Zanre et al., 2022). This report first reveals that cryptotanshinone inhibits human melanoma cell growth by reducing P21 and P53 protein expression.

The FOXM1 gene, which belongs to the FOX family, has been correlated with a negative prognosis in patients with cancerous tumors when it is overexpressed (Gormally et al., 2014). A study has shown that tanshinone IIA can reduce the expression of FOXM1 in gastric tumors, resulting in a rise in p21 and a decrease in PCNA and Ki-67 proteins, ultimately inhibiting the proliferation of SGC-7901 cells (Yu et al., 2017). Overall, tanshinone IIA could suppress the growth of gastric tumor cells by acting as a mediator of FOXM1.

### 3.3 STAT3 signaling pathway

The oncogene STAT3 has been extensively researched for the activation of its signal transduction pathway in tumorigenesis (Choudhari et al., 2007; Timofeeva et al., 2013). Given the important role of the JAK/STAT3 signaling pathway in tumor growth when IL-6 is activated in the microenvironment (Guo et al., 2012), studies on pertinent mechanisms have been undertaken. Wang et al. (2019b) found that tan I intervention remarkably prevented IL-6-induced JAK1/2 and STAT3 activation and concurrently suppressed the phosphorylation of JAK1/2 and STAT3. Additionally, this report implied that tan I could inhibit osteosarcoma cell growth and metastasis by restraining the interactions between STAT3 and its target genes Bcl-2, Cyclin B1, and MMP2. Additionally, another study revealed STAT3 may regulate SIRT3 production at the transcript stage, which further impacted levels of glycolysis-related proteins, including GLUT1, LDHA, and HK2. Cryptotanshinone was found to inhibit cell reproduction of ovarian cancer induced by glycolysis through suppression of the STAT3/SIRT3 pathway (Yang et al., 2018).

According to a study on gastric cancer, tan IIA reduced the survival of SNU-638, MKN1, and AGS cells in a manner dependent on dose and caused apoptosis by activating Bax and caspase 3 (Zhang et al., 2018). In the meantime, the level of phosphorylation of STAT3 was significantly inhibited and the same outcome was observed in the xenograft model. Notably, when SNU-638 cells were stimulated to overexpress STAT3, tan IIA was unable to inhibit cell reproduction. That is because STAT3 overexpression could counteract the influence of tan IIA on cell proliferation.

### 3.4 miR30b-P53-PTPN11/SHP2 axis

**FIGURE 4:**
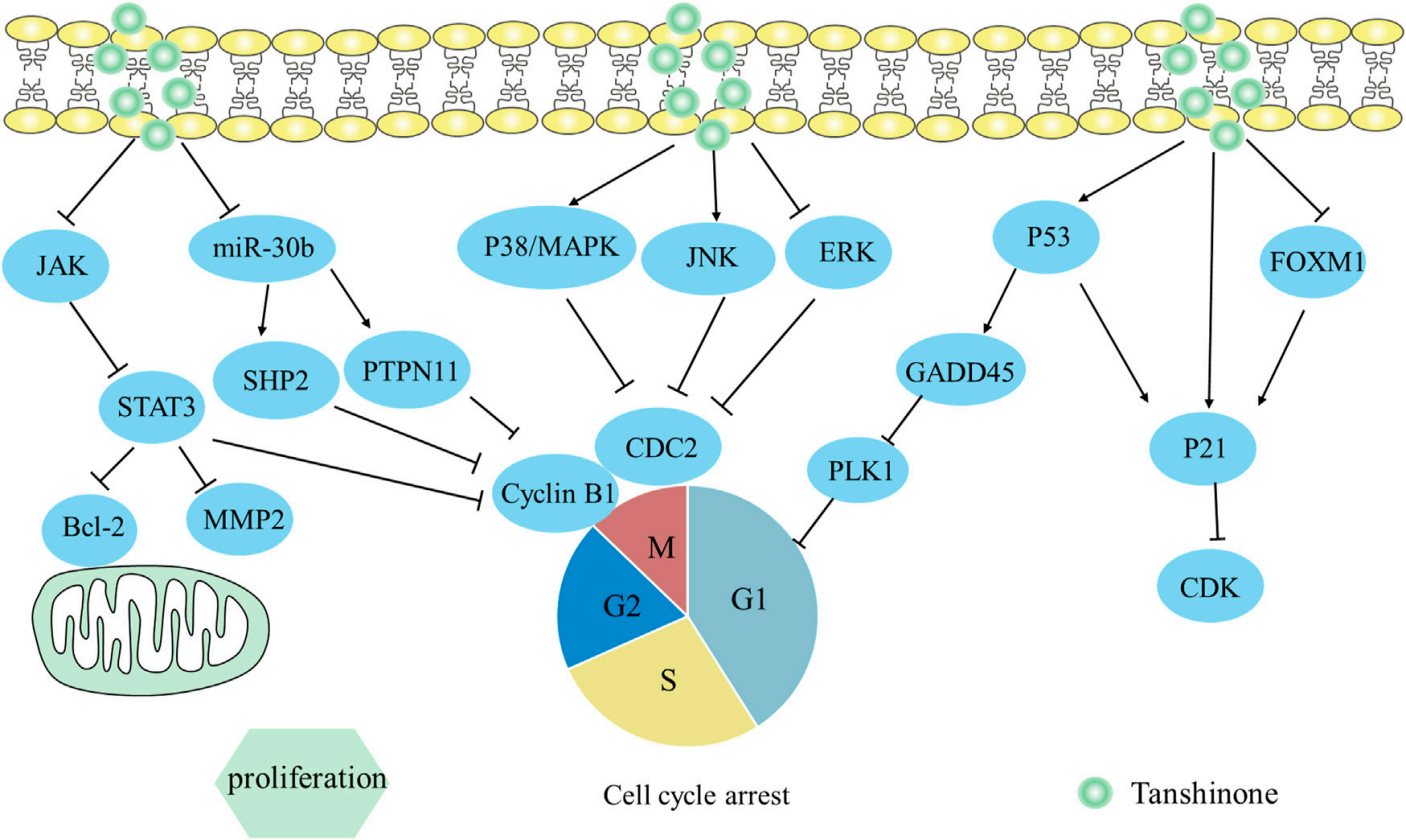
Mechanism of Tanshinone inhibiting proliferation in tumor cells. Contents are as follows. JAK/STAT3 restrains its target genes Bcl-2, Cyclin B1; miR30b-P53-PTPN11/SHP2 downregulate cell cycle-related proteins; Activate p38 MAPK and JNK MAPK pathways, but inactivate ERK MAPK pathway to downregulate CDC2 and cyclin B1; Activate p53-dependent GADD45A/PLK1 pathway; Activate p53/p21; Activate FOXM1/p21.

P53 was the sole transcription factor for the PTPN11 and the SHP2 protein was encoded by PTPN11 (Bentires-Alj et al., 2004; Han et al., 2015b). The research on hepatocellular carcinoma revealed that tanshinone IIA decreased the viability of HepG2 cells dose-dependently and inhibited proliferation with the inactivation of miR30b (Ren et al., 2017). Meanwhile, levels of P53 and P21 were notably increased, while Cyclin D1 and CDK6 were reduced in HepG2 cells. Notably, suppression of miR30b could trigger the expression of P53-PTPN11/SHP2 in HepG2 cells, and downregulation of cell cycle-related proteins, thereby inducing HepG2 cell cycle stagnation at G1/G0 checkpoints. Overall, Tanshinone IIA hindered the growth of liver carcinoma cells by blocking miR30b expression and stimulating the P53-PTPN11/SHP2 axis. The mechanism by which it inhibits proliferation is depicted in Figure 4.

## 4 Tanshinone prevents tumor cells from migrating and invading

Tumor cells possess the capacity for migration and incursion (Friedl and Wolf, 2003), which are the hallmarks of tumor malignancy (Zeng et al., 2019). Incursion requires active migration to occur beforehand (Entschladen et al., 2004), thus it is effective in impeding the migration and incursion of cancerous cells when treating tumors.

### 4.1 Tanshinone hinders tumor cells migration and invasion

#### 4.1.1 EMT

The activation of EMT enables cancerous cells to migrate and invade, assuming a pivotal role in cancer’s progression (Mani et al., 2008). EMT is a biological process where cells shift from epithelium to mesenchymal, and gain increased motility and invasive abilities by secreting MMPs. And it also can be induced by EGF and TGF-β1 (Puisieux et al., 2014). In accordance with a study, the level of EMT indicator E-cadherin was substantially decreased when induced by EGF and TGF-β1, while levels of Vimentin, N-cadherin, MMPs, and transcription factors (Snail, Slug, Twist) were significantly elevated in HepG2 cells (Zhang et al., 2019a). In that study, tan IIA can reverse EMT biomarker protein (MMP-2, E-cadherin, N-cadherin, vimentin, and Snail) changes in HepG2 cells, counteracting EMT triggered by EGF and TGF-β1. Additionally, elevated p-PI3K, p-Akt, and p-ERK expression were successfully reversed. This showed that Tan IIA hindered the process of EMT on liver cancer triggered by EGF and TGF-β1 by regulating the PI3K/Akt/ERK axis.

In colorectal cancer, the vitro study proved that tan IIA might hinder EMT in SW480 cells via reducing MMP-9 and Vimentin while raising E-cadherin, hence preventing cell migration and incursion (Zhang et al., 2016). Similarly, tanshinone derivatives inhibited prostate cancer cell metastasis by reducing VEGF-1 and MMP-9 protein expression (Wang et al., 2019a).

Additionally, Wang et al. (2023) observed that tanshinone derivative reduced Cavin-1 expression in NSCLC cells and suppressed the EMT process by decreasing levels of EMT indicators (N-cadherin, Vimentin, snail, slug, MMP2/7/9). In the meantime, tanshinone derivative efficiently reduced the ERK/Smad2 axis’s activation in normal cells and Cavin-1 transfected cell lines. This indicated tanshinone derivative impeded migration and incursion of NSCLC cells by suppressing the Cavin-1-mediated ERK/Smad2 axis.

#### 4.1.2 β-catenin

The molecule β-catenin is crucial for cancer cell motility, invasion, and angiogenesis, which is part of the Wnt/β-catenin pathway (Sui et al., 2015). β-catenin is liberated from the Wnt/β-catenin protein complex when activated by its upstream genes like TGF-β1 and HIF-1α (Jeong and Park, 2013). Subsequently, β-catenin can be combined with TCF/LEF and trigger the production of VEGF synergistically (Wu et al., 2015), leading to the promotion of angiogenesis and migration of tumors. Sui et al. (2017) discovered that tanIIA hindered VEGF-mediated angiogenesis under normoxic and hypoxic conditions. It worked by blocking different signaling pathways in each condition: TGF-β1/β-catenin/TCF3/LEF1 in normoxic conditions and HIF-1α/β-catenin/TCF3/LEF1 in hypoxic conditions. This implied that tan IIA potentially impeded colorectal cancer’s spread by blocking the β-catenin/TCF3/LEF1 signaling pathway via the suppression of TGF-β1, HIF-1α.

Another study in colon cancer revealed that tanshinone IIA impeded the incursion and migration of HC8693 cells by suppressing angiogenesis (Ma et al., 2018). In the meantime, the Wnt/β-catenin axis was inactivated, together with reductions in COX-2 and VEGF. Notably, the reduction of β-catenin can result in a decrease in VEGF expression and lead to block angiogenesis in tumor cells, thus affecting tumor cell growth and migration (Kim et al., 2016; Fu et al., 2017). Overall, tanshinone IIA suppresses the migration of colorectal carcinoma cells by blocking the COX-2-Wnt/β-catenin axis.

Yuan et al. (2020) discovered that tan IIA exerted an anti-invasion impact on gastric cancer, in a way that depended on time and concentration. During the study, levels of HMGB2, β-catenin, and various downstream molecules including c-myc, cyclin D1, N-card, and Vimentin were reduced simultaneously through the use of tan IIA intervention. Additionally, miR-874 overexpression had a negative effect on the HMGB2/β-catenin pathway, which suggested targeting miR-874 could be an important strategy for treating gastric cancer. This result indicated that tan IIA inhibited the spread and incursion of AGS, MGC-803 cells by the miR-874/HMGB2/β-catenin pathway.

#### 4.1.3 Hippo signaling pathway

The dysregulation of the Hippo pathway has the potential to cause tumor metastasis and oncogenic effects (Qiao et al., 2018). It is comprised of a principal kinase cascade that includes Mst1/2, and LATS1/2, as well as downstream transcription co-activators YAP or TAZ. Qian et al. (2018) discovered that tan IIA/IL-2 activated the Mst1-Hippo pathway and elicited INF2-related mitochondrial fission of SW480 cells, subsequently promoting apoptosis and inhibiting migration in colorectal cancer. This finding suggested a strong relationship between the Mst1-Hippo pathway and mitochondria.

Furthermore, YAP is a crucial component of the Hippo pathway. A study has been discovered regarding the relationship between SMAD7 and the Hippo/YAP axis in liver carcinoma (Ma et al., 2019). SMAD7 negatively regulates the Hippo/YAP pathway which could promote E3 ligase βTrcp expression to advance the degradation of YAP protein. Ma et al. (2019) observed that Tan IIA could boost E-cadherin and SMAD7 expression and reduce N-cadherin and YAP expression, which inhibited the metastasis and invasion of Bel-7404, SMMC-7721, and Bel-7402 cells. This indicated that tan IIA could impede the spread and incursion of liver cancer via inhibiting the Hippo/YAP pathway.

Qin et al. (2018) discovered that tan IIA therapy inhibited HuR translocation from the nucleus’s interior, causing a substantial downregulation of YAP which was highly expressed in cervix carcinoma. Meanwhile, the downstream target protein E-cadherin increased, while N-cadherin decreased, which weakened the capacity of HeLa and C33A cells to metastasize and invade. This indicated that tan IIA was capable of hindering cervical carcinoma cells from spreading and invading via decreasing the production of YAP.

#### 4.1.4 Skp2 signaling pathway

**FIGURE 5:**
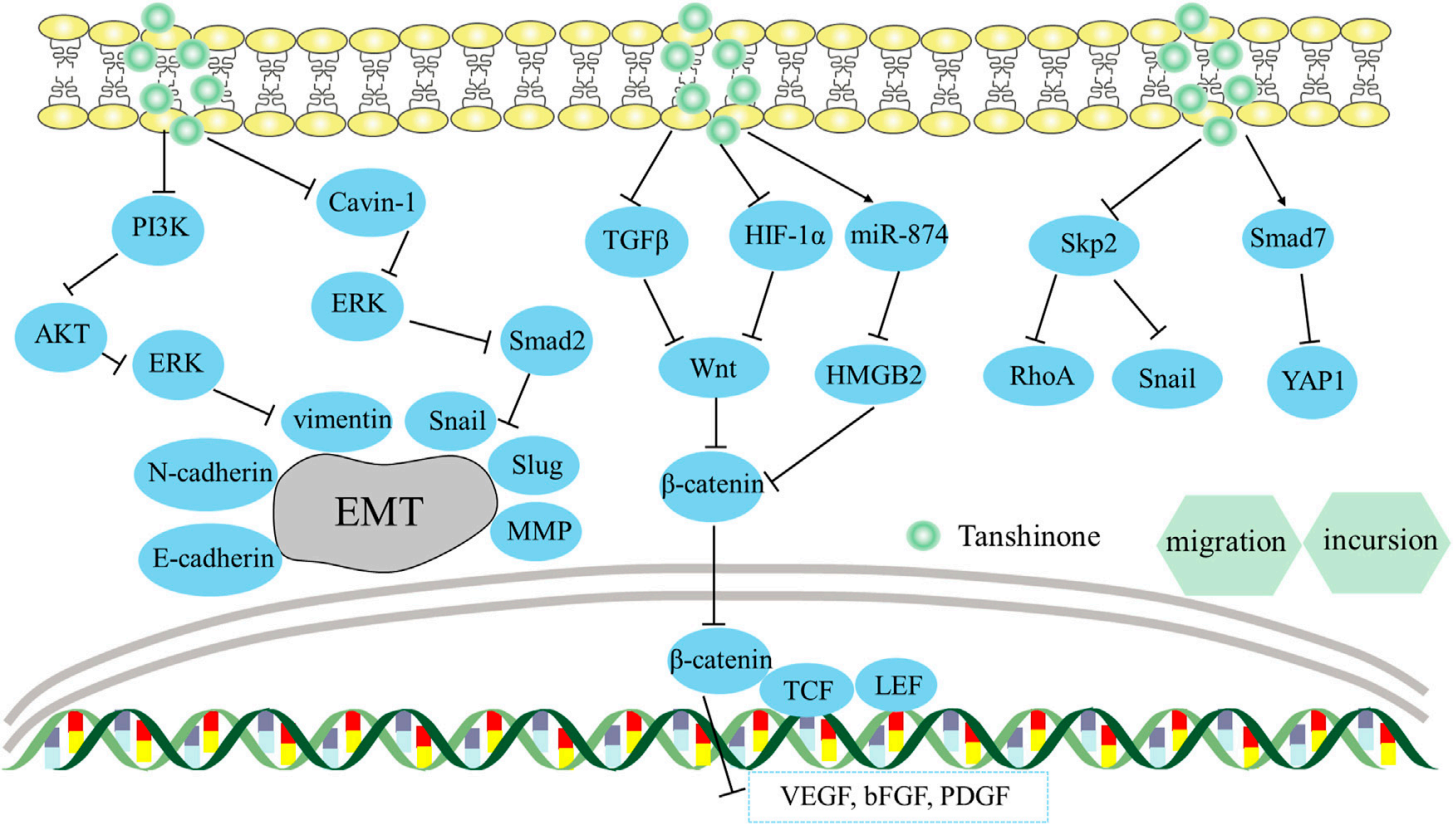
Mechanism of Tanshinone preventing migration and incursion in tumor cells. Contents are as follows. Hinder EMT via regulating the PI3K/Akt/ERK axis; Suppress the Cavin-1-mediated ERK/Smad2 axis; Block the β-catenin/TCF3/LEF1 axis via the suppression of TGF-β1, HIF-1α; Regulate miR-874/HMGB2/β-catenin pathway; Inhibit Skp2 and its downstream EMT genes’ expression, including RhoA and Snail; Boost SMAD7 levels and reduce YAP levels.

Skp2 is elevated in human cancer and may be crucial for the progression of cancer (Lin et al., 2017). Skp2 could accelerate the spread of cancer by regulating the proteins involved in EMT, such as MMP-9 and snail1 (Hung et al., 2010; Wei et al., 2013; Yang et al., 2014). Consistently, the Skp2 complex controls RhoA transcription, which is crucial for cancer migration (Chan et al., 2010). According to research, dihydrotanshinone I could reduce the expression of Skp2 in HCT 116 and HT29 cells, leading to lower levels of RhoA and snail1, ultimately inhibiting the spread of colon cancer (Lin et al., 2017). In addition, dihydrotanshinone I also exerted the prostate cancer’s ability to forestall metastasis by mediating Skp2 *in vitro*. Meanwhile, Skp2 and its downstream EMT genes’ expression, including RhoA and snail1, were inhibited following dihydrotanshinone I treatment in a manner dependent on concentration, which resulted in reduced invasiveness of PC-3 cells (Wu et al., 2017). These studies showed that inhibiting Skp2 could be a promising strategy for cancer treatment. Its particular mechanism for preventing migration and incursion is depicted in Figure 5.

### 4.2 Tanshinone inhibits tumor angiogenesis and permeability

Tumour angiogenesis helps tumors develop, invade, and metastasize. A recent study showed that the hypoxic microenvironment is the main mechanism that shifts tumors from avascular to angiogenesis (Huang et al., 2016). The hypoxic microenvironment stimulates HIF-1α in tumor cells, leading to higher expression of angiogenic factors like VEGF and bFGF, which promote tumor formation and vascularization. Conversely, Angiostatin and Endostatin hinder angiogenesis. Study data in lung cancer showed VEGF expression reduced while Angiostatin and Endostatin expressions elevated, implying that tanshinone IIA treatment decreased angiogenesis of tumor cells. In addition, COX-2 overexpression also triggers increased VEGF production, exerting a distinctly encouraging effect on angiogenesis. Zhou et al. (2012) found that tan II dramatically reduced COX-2 and VEGF levels in HCT-116 cells, blocking the angiogenesis of colorectal cancer.

Furthermore, HIF-1α expression is still a major marker for detecting cancer neovascularization (Kasai et al., 2018). Research revealed that tanshinone I could attenuate hypoxia-induced HIF-1α accumulation and phosphorylation of Tyr705-STAT3 in MCF-7 cells (Wang et al., 2015b). Subsequently, a decrease in VEGF secretion could prevent endothelial cell activation, exerting tanshinone I’s antiangiogenesis effects. Additionally, Zhou et al. (2020b) discovered that tan IIA reduced HUVEC proliferation, tube formation, and metastasis by decreasing HIF-1α, VEGF, and bFGF expression dose-dependently in HCT-116 cells, preventing angiogenesis in colorectal cancer. Taken together, these findings demonstrate that tanshinone-mediated HIF-1α emerges as an alternative cancer treatment.

Tumour blood vessels have abnormal morphology, resulting in permeable, immature, weak, and poorly perfused vessels. Tumour blood vessels’ permeable profile impedes the transportation of oxygen, immune cells, and therapeutic drugs, resulting in an acidic and hypoxic milieu, impaired medication delivery, and immune cell infiltration (Viallard and Larrivée, 2017). Zou et al. (2021) found that tan IIA lowered blood vessel permeability and improved colon cancer’s vascular integrity by targeting the Ang2-Tie2-AKT-MLCK pathway in transplanted HT-29 tumors. Overall, reverting premature and permeable blood vessels to normal blood vessels could become an efficient tumor treatment method.

### 4.3 Tanshinone suppresses the formation of metastases

Surgery is necessary for most solid tumors, however, it might lead to recurrent and metastatic lesions, hence it is vital to avoid metastases after surgery. Wang et al. (2012) found that tanshinone IIA can reduce hepatocellular carcinoma metastasis after palliative resection via normalizing VEGFR1/PDGFR-related vascular function. It did significantly raise microvessel integrity throughout this procedure. This finding suggested that proangiogenic “vessel normalizing” treatments may block the formation of metastases and improve patients’ survival. Besides, in colon carcinoma, tanshinone IIA reduced uPA, MMP-2, and MMP-9 and increased TIMP-1, and TIMP-2 dose-dependently to limit the formation of metastases *in vitro and in vivo* (Shan et al., 2009). Furthermore, tanshinone lowered HMGB1, a metastasis-related gene, limiting the metastases of gastric cancer (Nishiguchi et al., 2019). Therefore, tanshione may be an option for blocking the formation of metastases.

## 5 Tanshinone increases the sensitivity of radiation and chemotherapy

Chemotherapy and radiotherapy are known to cause negative effects and increase drug resistance (Sekeres et al., 2021). A relevant study on lung cancer found that H358-IR and H157-IR cells of radio-resistance had notably lower levels of Phosphoribosyl pyrophosphate aminotransferase (PPAT) after being treated with tanshinone I. This treatment also led to raised Caspase 3 and Caspase 8 levels, more DNA damage, and a notable increase in apoptosis (Yan et al., 2018). This finding suggests that tanshinone I has the potential to improve cell sensitivity to radiotherapy by reducing the levels of PPAT, which is known to promote tumor growth in cells.

The obstacles of chemotherapy are closely related to multidrug resistance (MDR). The primary cause of MDR is the increased levels of ABC transporter proteins, such as MRP1, P-gp, and BCRP, expressed in cancerous cells (Amawi et al., 2019). Cancer cells that are resistant to chemotherapy may overexpress export transporters, which can transfer chemotherapeutics away from cancer cells, and decrease their cellular storage and effectiveness for treatment. According to a study, tan IIA can reduce the expression of MRP1 in gastric carcinoma cells, which causes SNU-719R cell cycle stagnation and apoptosis (Xu et al., 2018). Co-treatment of tanshinone IIA and doxorubicin promoted apoptosis by modulating the expression of Bcl-2, Bax, and P53, and triggered G2/M stage cell cycle stagnation by modulating levels of cyclin B1 and CDK1. There was also a study reporting that cryptotanshinone and dihydrotanshinone reversed doxorubicin and irinotecan resistance in SW620 cells of colon cancer by decreasing the level of P-gp mRNA and blocking P-gp ATPase activity (Hu et al., 2014). Taken together, reductions of MDR expression and function have a significant impact on chemotherapy.

**FIGURE 6:**
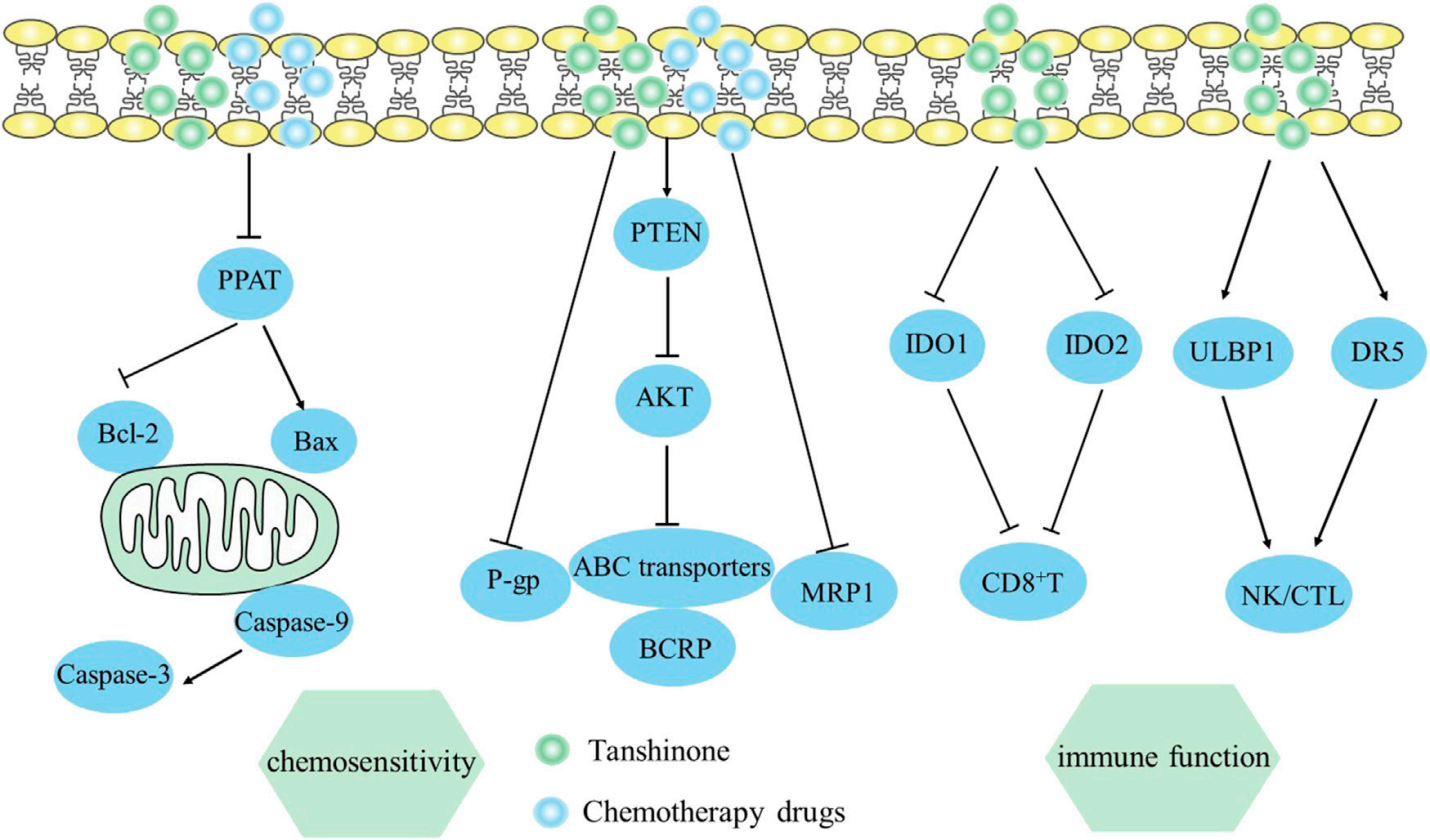
Mechanism of Tanshinone increasing sensitivity of chemotherapy and hindering immune evasion in tumor cells. Contents are as follows. Sensitivity of chemotherapy: Increase PTEN and block the PI3K/AKT axis expression to reduce ABC transporter proteins. Immune evasion: Increase ULBP1 and DR5 to enhance NK/CTL activities; Suppress IDO1 and TDO2 to elevate CD8^+^ T cells.

It was also reported that tanshinone IIA may be effective in overcoming doxorubicin resistance in MCF-7 cells of breast cancer, which was achieved by blocking the PI3K/AKT axis and reducing levels of ABC transporter proteins, including MRP1, P-gp, and BCRP (Li et al., 2019). Moreover, Tan IIA’s intervention prevented AKT activation due to the increase of PTEN expression. *In vivo* study also revealed that tan IIA improved the effectiveness of doxorubicin in treating AGS cell xenograft tumors of nude mice (Li et al., 2019), while simultaneously mitigating negative adverse effects, such as loss of weight, myelosuppression, and renal toxicity. The mechanism by which it enhances the body’s response to chemotherapy is viewed in Figure 6.

## 6 Tanshinone hinders tumor immune evasion

Multiple immune evasion mechanisms in cancer cell proliferation contribute to their ongoing occurrence and development, making immune response crucial for tumor treatment (Li et al., 2016b). The study in lung cancer found that tanshinone IIA treatment significantly increased CD4^+^ and CD4^+^/CD8^+^ levels *in vivo*, as well as NK cell activity, further improving immune function and strengthen anti-tumor effect (Li et al., 2016b). Similarly, Sun et al. (2020) found that tanshinone IIA might improve NSCLC cell sensitivity to NK cell-mediated lysis by increasing ULBP1 and DR5, demonstrating tanshinone IIA’s potential in NK cell-based cancer immunotherapy. Additionally, mice treated with tanshinone and radix astragali enhanced CD4^+^ and CD8^+^ percentages and NK/CTL activities *in vivo*, affecting immune system functions in melanoma (Wu et al., 2016). Zhang et al. (2022) indicated that tanshinone IIA sulfonate might be used as an immunotherapy for colorectal cancer by suppressing IDO1 and TDO2 that promote tumor immune evasion. Ultimately, tanshinone IIA sulfonate enhanced tumor immunotherapy by decreasing Tregs and elevating CD8^+^ T cells respectively. Overall, tanshinone enhances the cytotoxicity of CD8^+^ T cells or NK cells, avoiding tumor immune escapes. The mechanism by which it hinders tumor immune evasion is viewed in Figure 6.

## 7 Summary and perspective

Tanshinone has garnered significant focus due to its various pharmacological effects. Several studies have demonstrated its ability to fight tumors through diverse molecular mechanisms such as apoptosis, ferroptosis, pyroptosis, reproduction, migration, incursion, chemosensitivity, and immune evasion. Regardless of multiple studies, further research is still required to fully comprehend the tumor-fighting mechanism of tanshinone. There are techniques such as network pharmacology and molecular docking that can be used to investigate potential mechanisms. However, it is also vital to conduct experimental validation in order to provide more reliable references for clinical workers.

Moreover, tanshinone is a crucial component in chemotherapy, and its impact on a patient’s prognosis is significant. When used alongside other chemotherapy medications, tanshinone can enhance their effectiveness and improve patient outcomes. However, one limitation of tanshinone is its limited bioavailability when taken orally. Thus, researchers are working on developing new formulations to improve its pharmacokinetic properties. To conclude, more research is needed to fully comprehend the potential benefits of tanshinone for clinical use in the future.
